# Editorial in This Issue

**DOI:** 10.1590/S1677-5538.IBJU.2015.04.01

**Published:** 2015

**Affiliations:** Divisão de Urologia do A.C. Camargo Cancer Center, Fundação A. Prudente, São Paulo, Brasil

In this issue of **International Brazilian Journal of Urology**, in the Difference of Oppinion, there is a highly controversy subject: The managing of bulbar stenosis Dr. Santucci, from Detroit, favors the buccal urethoplasty against the complications of substitution uretroplaty and the bad results of internal urethtrotomy; on the other hand, Drs. Siegel and Morey justify why the excision and primary anastomotic urethroplasty is considered the gold standard in Dallas. A third vision came from Drs. Cavalcanti and FIeder, from Rio de Janeiro, which suggest that in selected cases (<1cm lesions, without spongiofibrosis, or in poor surgical risk patients) the internal urethrotomy followed by office dilatations may be an attractive alternative. On the page 623, a *Review article* brings actual information regarding the the plead meshes devices avialble for vaginal prolapses surgeries. This is fundamental, since at the same time the use of the mesh became popular, the posts about their complications and malpractice questions are so common.

There are reports and concerns about the risks of high intraocular and intracranial pressures during robotic assisted radical prostatectomy (RALP), due the long time of Trendelemburg position and pneumoperitoneum during the surgery. The Connecticut's group (page 661) analyzed retrospectively the post RALP outcomes of 40 patients with pre existing retinal or central nervous system comorbidities. There were no more Retinal or SNC complications in comparison with the remaining 1828 patients.

The use of use of pre-procedural computed tomography angiography, can helps to know better in advance the renal vascular anathomy, reducing the surgical bleeding in miny percutaneous surgery (page 690). Despite the use of External Shockwave litothripsy has decreased for the ureteral stones management, it is yet a valid alternative. Yazici et al. reported the independent prognostic factors for stone fragmentation in proximal and distal ureteral stones, in a cohort of 204 patients. (page 677). Higher body mass index (BMI) patients had lower stone fragmentation in distal calculi. Higher BMI patients were also investigate in this journal number about the their rates for surgical complications after nephrectomy (page 697) and about the effectiveness of transobturator mid urethral versus mini slings in this population(page 714), reflecting the general interest in the repercussion of progressive obesity in the world.

The modern lifestyle and environmental conditions may be among the reasons of the reduction of semen quality in Brazilian patients, according Borges Jr. et al. (page 717). At page 773, the protective effects of dexamethasone and vitamin E in the testicular parenchyma oxidative damage of a varicocele induced rat model are shown.

*Pediatric Urology*: A group from Bahia reported the acute and the late urodynamics effects in children with idiopathic overactive bladder underwent parasacral transcutaneous electrical.

